# The ‘Aachen Falls Prevention Scale’ - development of a tool for self-assessment of elderly patients at risk for ground level falls

**DOI:** 10.1186/s13037-014-0055-0

**Published:** 2015-02-14

**Authors:** Hans-Christoph Pape, Ulrike Schemmann, Juergen Foerster, Matthias Knobe

**Affiliations:** Department of Orthopaedic Trauma, University of Aachen Medical Center, 30 Pauwelsstreet, 52074 Aachen, Germany; Division of Physical Therapy at University of Aachen Medical Center, Aachen, Germany; Harald Tscherne Lab for Orthopaedic Trauma, University of Aachen Medical Center, Aachen, Germany

**Keywords:** Orthogeriatrics, Co-managed care, Fractures in the elderly, Geriatric trauma center, Fall prevention, Balance assessment

## Abstract

**Background:**

The incidence of falls in the elderly population is difficult to determine and therefore potentially underestimated. Screening algorithms usually have in common that the evaluation is undertaken by trained individuals in a hospital setting. This leads to the inclusion of a high proportion of low-risk people and a waste of resources. It would be advantageous to pretest the individuals at risk in their own environment using a simple self-assessment approach.

**Methods:**

The consensus process of our group of clinicians and physical therapists included: 1. a preparative literature review about risk profiles and assessment tools for ground level falls; 2. a selection of appropriate questions that cover all health aspects involved in an increased risk for falling; and 3. a selection of a simple physical test that can be used at home without the need of a health care professional. We thus searched to develop a scale that can be used by older citizen at higher risk of falling. The current manuscript summarizes the results of this review, consensus and selection process.

**Results:**

The literature search was undertaken between March and August 1, 2013. The selection process for the questions used (Part I) lasted between March 2013 and January 2014. Among all tests evaluated the 20 second standing test (Part II) was deemed to be safe to be performed even by an individual at risk for a fall, as it closely resembles activities of daily living. The `Aachen Falls Prevention Scale` finally uses a self-assessment tool grading falls risk on a scale of 1 to 10 by the individual itself after completion of Part I and Part II. In summary, we present a scale that might offer a self-assessment option to improve the measures of falls prevention pass for elderly citizens.

**Conclusions:**

The introduction of the `Aachen Falls Prevention Scale` which combines a simple questionnaire with a safe and quick balance tool, meets the criteria to identify whether or not a balance problem exists – the first step in evaluation of falls risk. Further studies will have to assess the ability of an individual to estimate his or her individual falls risk on a longitudinal basis and possibly trigger the necessity for the assessment by a physician.

## Background

Multiple studies have described the demographic changes in western societies. An increasing number of traumatic events are associated with these changes [1-6]. These are accompanied by a substantial increase in health care costs, both for in hospital and out patient treatments.

The incidence of falls in the elderly population is difficult to determine and therefore potentially underestimated. Even in the cognitively fit patients, reconstructing the chain of events is demanding [7]. In this light, it is important to have a profound knowledge about the mechanisms of falls in the geriatric population.

The patients’ “natural habitat” seems to be directly related to the individual risk for a low energy fracture. There is only a limited amount of studies that investigated the risk of a fall and those were performed in health care facilities, e.g. hospitals or nursing homes. Despite the variety of potential risks in the outside world, older people preferentially fall at home. This might attributable to the disproportionately large amount of time staying indoors and a higher level of confidence and carelessness [8]. Vlaeyen et al. recently provided new information by doing a home based video analysis of falls in older patients. Their results clearly indicate that the majority of falls occur in the morning and in the evening, rather than during the day or at night [2]. There is a positive correlation between the number of risk factors and the probability of actually falling [8]. Previous falls, strength, gait and balance impairments, and use of specific medications range amongst the strongest predictors [9]. By identifying individuals with a high risk of falling, targeted fall prevention interventions could be directed at those most likely to benefit from them. This screening process is distinct from the more intensive assessment procedures that are used to identify potentially modifiable risk factors in multifactorial fall prevention programs [10]. The main purpose of a less complex first screening tool is the identification of a potential balance problem [11-14]. Simple screening questions have been reported to perform as well as more complex screening tests in predicting who will fall [10]. The American Geriatric Society/ British Geriatric Society guideline therefore suggests that all older individuals should be assessed regarding the incidence and number of falls in the last year. Furthermore, the evaluation should include a detailed physical examination and a timed performance test. Those found by this screen to be at higher risk should be given more intensive assessment and intervention [10,12]. These screening algorithms usually have in common that the evaluation is undertaken by trained individuals in a hospital setting. This leads to the inclusion of a high proportion of low-risk people and a waste of resources while at the same time the omission of a large proportion of people at high risk limits the potential reduction in falls achievable [10].

In this light, it would be advantageous to pretest the individuals at risk in their own environment using a simple self-assessment approach.

The purpose of the consensus process of our group of clinicians and physical therapists was threefold:

1. to review the available literature about risk profiles and assessment tools for ground level falls
2. to select appropriate questions that cover all health aspects involved in an increased risk for falling
3. to select a simple physical test that can be used at home without the need of a health care professional and without having an increased risk of a fall while using it.

We thus searched to develop a scale that can be used by older citizen at higher risk of falling. The current manuscript summarizes the results of this review, consensus and selection process. It was designed to allow for a prospective test of ease of use, validity and specificity.

## Materials and methods

### Prerequisites for feasibility

The author panel decided on the following prerequisites for the data selection; availability and completeness of data, worldwide applicability, sensitivity and specificity to describe the individual at risk of a ground level fall. The following a-priori assumptions were applied:

### A-priori assumptions by the author panel

1. The basis for the new scale should allow every individual to assess his/her own risk at defined time points. No second individual should be required to perform the assessment.
2. The assessment should be feasible by any layman. No physician’s assistance should be involved.
3. Any risk for falls should be covered, if caused by medical, neurologic and orthopaedic diseases.
4. There should be a combination of a questionnaire and a practical part.
5. The risk of a fall of the elderly patient while performing the practical part of the assessment should be minimal.
6. The assessment should be reliable and repeatable to allow for longitudinal comparison.
7. It was agreed that feasibility is a key factor for the success of the questionnaire. Therefore, any assessment exceeding 10 questions was eliminated.

### Preparative literature review

A review of the literature was performed on the available assessments for falls and injuries. The following media was searched:

1. MEDLINE, EMBASE, PsycInfo, CINAHL, and Social Science Citation Index
2. The World Wide Web
3. Publications from medical insurance companies
4. Recommendations for falls prevention published by multiple institutions, such as the WHO and geriatric societies

All original articles were included if published within Jan 1, 1940 and Dec 31, 2013. No language restrictions were applied. This review served to determine pertinent parameters and cut-off values for the individual ‘at risk’ for a fall. We inspected the reference lists of all eligible studies to further identify any potentially eligible studies that were cited by them.

The following eligibility criteria were used [10]:

1. Prospective cohort studies that evaluated one or more screening tests inclusive reviews of them
2. Studies and reviews that evaluated multiple impairments and conditions predisposing to falls
3. Inclusion of elderly people living in the community or substantially independently
4. Falls were recorded prospectively.

Data were extracted by two reviewers independently, and discrepancies were resolved by discussion.

For the practical part, all routine tests used by geriatricians - such as the Tinetti gait, balance, or mobility scales (also referred to as the Performance Oriented Mobility Assessment, POMA), the Timed Up and Go Test (TUG test), and other tests evaluating stance, walk and balance were considered as well. However, the panel felt that most of them impose an unacceptably high risk when used without the help of a health care expert.

All potential questions were presented to a board of potential users for assessment of feasibility, understandability and ease of use. The selection process for the questions used lasted between March 2013 and January 2014 and is listed in Table 1.

**Table 1:** **Time course of the consensus process and development of the `** ***Aachen Falls Prevention Scale`***

**Pre-Development and scientific discussions**	
-	Kick-off session at the AO Geriatric Course, Aachen 2013
-	Pre course meeting for AO Geriatric course in Zurich, Switzerland, 2011, Milan
**In-person discussions (Oct 15, 2013 – Jan 10, 2014)**	
-	Meeting to discuss the composition of the expert panel group (Berlin, German Congress of Orthopaedics/Trauma, DKOU 2013)
-	Pre-circulation of a preliminary time line prior to DKOU
**Literature review (July 1, 2013 – September 10, 2013)**	
-	Assessment of Pub med searches, evaluation of falls tests published on websites and information published by insurance companies
**Selection of tests for the practical part of the assessment**	
-	In person panel meetings between Oct 5 and Dec 21, 2013)
**Consensus meeting, Jan 10, 2014**	
-	Approval of 10 questions and the practical test

## Results

The kick-off meeting was held during the AOTrauma Seminar `Geriatric Trauma and Aging Bone` (March 15/16, 2013). The literature search was undertaken between March and August 1, 2013. All references were sequenced and sorted by the authors.

Between August 1, 2013 and January 10, 2014 multiple in person meetings of the panel of authors were undertaken (Table 1). The electronic search yielded 4,356 citations. Of these, 195 were selected for further consideration based on their title and abstract and full reports were obtained. The search of the reference lists of the review articles and all selected studies yielded an additional 19 potentially eligible studies. Out of these 214 publications, websites and summaries, 56 met the inclusion criteria and were assessed. The most common reasons for exclusion were that the study included an ineligible population (usually hospitalized participants), falls data were not collected prospectively, or the required data could not be extracted. Among the selected publications, the following criteria were applied.

### Issue of physician based assessment

Among these, we used multiple questions to assess the patient. Those that require physician assistance and those that require the expertise of a geriatrician were ruled out to allow for patient self-assessment.

### Value of practical testing

26 tests were evaluated. Among these, 15 appeared to be feasible. 12 require assistance by a health care professional and 7 could be performed alone.

Among these the 20 second standing test was deemed to be safe to be performed even by an individual at risk for a fall, as it closely resembles activities of daily living.

### Selection of questions

All questions addressed in this score sheet have been evaluated by independent committees or researchers. They all have in common that they require a normal state in mind and cannot be performed in case of dementia.

The final meeting to reach consensus about the ´Aachen Falls Prevention Scale´ was held on January 10, 2014.

## Discussion

Time constraints, competing demands, and inadequate reimbursement pose a challenge to incorporating fall prevention into practice [15,16]. Evidence-based fall risk assessment and management becomes feasible and effective with the implementation of screening instruments, acknowledgement of reciprocal effects of competing conditions and a resilient and confident network of members of the health care team [9]. A close cooperation between multiple specialties represents a key factor in the prevention and treatment principles of elderly patients at risk of falls [3,7-11]. The causes of falls can be multifactorial and may derive from cardiac arrhythmias, cerebrovascular, neurologic causes, or are well described to require the care of an ear, nose and throat physician. Most of the available falls assessment scales therefore use a variety of questions in order to demonstrate a well-rounded picture of the patient at risk [9-11].

Runge et al. reviewed the determinants of musculoskeletal frailty and the risk of falls individuals with older age. They suggest five musculoskeletal tests to be relevant determinants of the risk of a fall with self-selected gait velocity being the single best parameter [13]. However, none of these tests can be safely used for self-assessment or at home. A history of falls as well as gait or balance disabilities seem to be strong and reliable predictors of future falls. Simple screening questions might therefore perform well enough in predicting falls, and little or no additional value may be gained by performing a complex screening test [10,12,14]. In conclusion, no further assessment is required if patients negate more than one noninjurious fall and do not report any difficulties with walking or balance [9,12]. Assessment tools for gait and balance are straightforward, self-contained, fast to apply and can apparently be used to predict the risk of falling [17]. However, there is a lack of evidence that any of the available screening tests is clearly useful for identifying fallers [10]. Evaluation of these tests has mostly been performed in single studies or in multiple but diverse and incomparable studies in terms of sample size or study design [10].

The most frequently evaluated tool is the Tinetti balance, gait, and mobility scales. However, different versions of the test were used by different studies [18]. Disadvantages of the scale include difficulty to assess many of the items on a 3-point scale and its poor specificity. Despite being widely used in gerontology, the gait section is seldom used [19]. The Berg Balance Scale (BBS) is easy to use and shows strong internal consistency and a high intra- and interrater reliability. On the other hand, the sensitivity is only poor to moderate [20]. The Timed Up and Go Test (TUG) is the most frequently recommended screening test for mobility. It uses agreement in stop-watch durations instead of rating scales and thus probably making it the most reliable test [11,19]. In line with several other clinical tests, it is poor at identifying the affected subcomponents of gait and balance [11,21]. The Tandem Stance is reported to have poor discriminatory ability and sensitivity but good specificity [22,23]. One-leg stance duration with eyes closed is often too difficult and variable to serve as a useful clinical test so that the eyes open version is generally used. Disadvantages include the difficult nature of the test and its lack of evaluating dynamic balance control [11].

In this light, we think the introduction of the combination of a simple questionnaire with a safe and quick balance tool, such as the 20 seconds standing test (`Aachen Falls Prevention Scale`) meets the criteria to identify whether or not a balance problem exists. An elderly patient who fails this quick balance screen, should have a more complete balance or gait evaluation by a physician or occupational therapist. Balance disorders can have serious consequences for the social function. Fear of falls leads to activity restriction and social isolation. However, in the light of lacking evidence on the accuracy of screening tools for predicting falls risk [10], the `Aachen Falls Prevention Scale` finally uses a self-assessment tool grading falls risk on a scale of 1 to 10 by the individual itself after completion of Part I and Part II. This summarizes the felt falling risk resulting from the multidimensional evaluation of risk factors and the balance control.

### Co-managed care in the case of a fall

If a fall requires hospitalization or even surgical intervention, the co-managed care concept foresees initial geriatric assessment. This implies a common treatment plan both for the fracture and the comorbidities. During the hospital stay, common ward rounds between geriatricians, orthopaedic trauma surgeons, social workers and specialized nurses have become the rule, at least in geriatric fracture centers [4]. In some areas even hospitals were built that focused on co-managed care [5]. Some studies document that the implementation of a standardized orthopaedic-geriatric care plan improves the outcome of proximal femur fractures [6,24]. It appears though, that the current literature about prospective studies regarding interdisciplinary treatment in hip fractures is difficult to prove and inconsistent results were shown [25]. Furthermore, there is no consensus on efficacy of the different ways of cooperation between orthopaedic surgeons and geriatricians [26]. Using multidimensional assessment strategies, it is difficult to identify the specific patient cohorts who could potentially benefit from this complex cooperation [27]. In addition, attention should be paid to cost-value ratios [27]. Several countries in Europe have begun a process of certification in order to improve the issues of co-managed care. Among others, our group has contributed to the selection process and the standardization of criteria for geriatric trauma centers [28].

## Limitations

This report has several drawbacks. Firstly, although the scale presented has been developed by a panel of health care experts, it lacks verification at the current stage. However, the authors felt that the need for a self-assessment tool is urgent and that the assessment should rather be performed in a large population than to publish a study that might potentially be biased through a type two error. Therefore, the `Aachen Falls Prevention Scale` is currently been evaluated in cooperation with one of the largest insurance companies in Germany. Secondly, the selection of questions is non-specific and does not relate to a specific disease. Thirdly, it does not yield to a diagnosis of a disease and its possible end result appears to be the decision of the individual to see a physician for further assessment.

Among the possible advantages is the fact that the assessment covers all aspects of diseases related to falls and can be performed independent of a physician. It supports the independent life of older individuals that feel responsible for their health condition. Moreover, the scale is repeatable as required and as felt by the older citizen. Furthermore, it supports the individual decision making as to whether a physician is seen and thus promotes the self-decided individual.

In general, many programs have focused on fracture prevention through specialized training. Among these are offers by physical therapists to train with walking aids. These should be performed on a regular basis and as a result, many places offer “drivers licenses for walkers”. These programs have to be managed by health care professionals in order to cover safety issues.

In summary, we present a scale that might offer a self-assessment option to improve the measures of falls prevention pass for elderly citizens. Further studies will have to assess the ability of an individual to estimate his or her individual falls risk on a longitudinal basis and possibly trigger the necessity for the assessment by a physician (Table 2).

**Table 2:** **‘Aachen Falls Prevention Scale’**

**Part I**		
*Self-questionnaire (10 questions, one point per question answered ‘yes’, 10 points max.):*		
	Yes	no
Do you have problems with hearing or vision?		
Do you feel unsafe or have you been falling recently?		
Are you afraid of falling?		
1. Do you take medication for sleep, cardiac problems, diuretics, or sedatives?		
2. Do you loose urine or stool involuntarily?		
3. Do you have memory problems?		
4. Do you feel lonely at times and think that your life is without value?		
Do you use a walking aid on a regular basis?		
5. Do you suffer from Parkinson’s, Arthritis or Rheumatism?		
6. Are there many traps that might cause a fall in your home?		
**Part II**		
*Self-Test with your partner*		
Stand freely, do not lean or hold on anybody, measure the time until you have to do a corrective action with your arm, upper body or lower extremity.		
**Standing test**		
Successfully completed: 20 seconds or more		
Failed: less than 20 seconds		
	Yes	no
**Conclusion and self-assessment:**		
How would you grade your falls risk on a scale of 1 to 10 (10 … max. risk)?		
If you score 5 points or worsening within the last weeks we recommend that you contact a physician for further assessment.		

## Conclusion

The current compilation of questions appears to represent a rounded number of questions that cover all areas of diseases that can contribute to an increased risk of falls. The development has been performed in a process including multiple steps. Yet, the set of questions have to be assessed in terms of reliability and accuracy. Therefore, a separate analysis will be required for verification. The introduction of the `Aachen Falls Prevention Scale` which combines a simple questionnaire with a safe and quick balance tool, meets the criteria to identify whether or not a balance problem exists – the first step in evaluation of falls risk. Whether or not making the second move and see a physician for complex falls risk evaluation is in the responsibility of the elderly patient.
